# Exploring chest wall deformities in childhood and adolescence: insights from a case-control study

**DOI:** 10.1186/s12887-024-05199-8

**Published:** 2024-11-06

**Authors:** Fedli Emre Kılıç, Osman Küçükkelepçe, Celal Varan, Hüseyin Tanrıverdi, Furkan Bakırhan

**Affiliations:** 1 Pediatrics, Adıyaman University, Adıyaman, Turkey; 2 Public Health Department, Adıyaman Provincial Health Directorate, Eski Saray District Sağlık Ocağı Street, Merkez, Adıyaman, 02100 Turkey; 3 Pediatrics, Adıyaman University, Training and Research Hospital, Adıyaman, Turkey

**Keywords:** Calcium, Children, Vitamin D, Pectus Carinatum, Pectus Excavatum

## Abstract

**Background:**

Chest wall deformities, though relatively uncommon in childhood and adolescence, significantly affect aesthetic perception and can impact cardiac and respiratory function. This study aims to compare individuals with pectus deformities to a healthy control group, shedding light on the condition’s etiology and prognosis.

**Method:**

Conducted as a case-control study between 2020 and 2022, the research included a case group of 71 patients with pectus excavatum or pectus carinatum who were followed up at the Pediatric Cardiology outpatient clinic. The control group consisted of 80 children without chronic diseases. Researchers retrospectively reviewed patient files, recording demographic data, echocardiography findings, and serum levels of calcium, phosphate, vitamin D, and alkaline phosphatase.

**Results:**

Patients with chest wall deformities exhibited significantly lower serum levels of vitamin D, calcium, and phosphorus compared to those without deformities. Males constituted 77% of the case group, with 15.5% exhibiting abnormal echocardiogram results, with mitral valve anomalies being most prevalent.

**Conclusion:**

While pectus deformities may lead to cardiac or respiratory issues, patient concerns often center around aesthetics. Unlike typical studies focused on surgical techniques and postoperative patients, our study focused on all diagnosed patients. Findings emphasize the importance of monitoring calcium, phosphorus, and vitamin D levels in pectus patients to manage surgical risks and facilitate recovery.

## Introduction

Chest wall deformities are characterized by the abnormal positioning of the sternum, either protruding forward or receding backward, due to irregular development of the costal cartilages. The incidence in the general population is estimated at 1% [1]. Chest wall anomalies are typically diagnosed when children start school, but they generally begin to cause clinical symptoms during the transition to adolescence [2]. In symptomatic cases, cardiological and respiratory complaints are common. Surgical repair indications vary by center, focusing on defect anatomy, physiological impact, and patient motivation [3].

Pectus excavatum (PE), the most common chest wall abnormality, is characterized by the depression of the sternum and costal cartilages. It occurs in approximately one in every 400 live births and is five times more common in males [4]. About 10% of cases are associated with Ehlers-Danlos or Marfan syndrome, while the rest occur sporadically. Although the exact etiology is not fully understood, it is believed to result from excessive growth or flexibility of the costal cartilages [5].

Pectus carinatum (PC) is the most common chest wall abnormality in children after PE. It is characterized by the forward protrusion of the sternum and is observed in approximately one in 1,500 live births. The condition is more prevalent in males and is often associated with syndromes such as Noonan, Poland, and Marfan [6]. While the exact cause is not fully understood, it is believed to result from excessive cartilage growth [3]. Symptoms like shortness of breath and chest pain are rare, with the primary concern being the aesthetic appearance [7].

Less commonly, chest wall deformities include pectus arcuatum and Poland syndrome. Pectus arcuatum is often associated with mitral valve prolapse, while Poland syndrome is characterized by the unilateral underdevelopment of the pectoral muscles [8, 9]. These syndromes, along with others besides PE and PC, account for 3–5% of all chest wall anomalies [10].

In the present study, various parameters were compared between known cases of PE and PC, and a control group without any known cardiological or metabolic diagnoses. Data that could influence the etiology or prognosis of chest wall deformities were evaluated in the affected group.

## Material and method

The study, conducted as a case-control study at Adıyaman Training and Research Hospital in Adıyaman, Turkey, spanned from 2020 to 2022. The case group comprised 71 patients with PE or PC, referred to the Pediatric Cardiology outpatient clinic from the General Pediatric outpatient clinic in the city and its districts. Children with diagnosed metabolic or connective tissue disorders, such as Ehlers-Danlos syndrome, Marfan syndrome, and other collagen-related disorders, were excluded from the case group. The control group consisted of 80 children with no history of chronic diseases, such as diabetes, vitamin deficiency, or metabolic and orthopedic conditions.

Patient files were reviewed retrospectively, and data were recorded by the researchers. Along with demographic characteristics such as age and gender, Echocardiography(ECHO) findings, and serum levels of calcium (Ca), phosphate (P), vitamin D, and alkaline phosphatase (ALP) were evaluated. In the echocardiographic evaluations performed by a pediatric cardiology specialist, certain valve anomalies such as tricuspid regurgitation, which are commonly observed in childhood, were not considered as echocardiographic abnormalities if they were within physiological or near-physiological limits.

### Ethical approval

Ethics committee approval for the study was obtained from the Adıyaman University Non-Interventional Research Ethics Committee (Decision No. 2022/8–19, dated 15.11.2022). Due to the retrospective nature of the study, participant consent was not required. However, written permission to use data from patient files was obtained from the relevant hospital. The study was conducted in accordance with the principles of the Declaration of Helsinki.

### Statistical analysis

The data were recorded in the SPSS 26.0 program, which was also used for error checks, tables, and statistical analyses. Descriptive statistics are expressed as numbers and percentages. Continuous variables are presented as mean, standard deviation, and median values. The t-test and Mann-Whitney U test were used to analyze relationships between continuous variables. Relationships between categorical variables were examined using the Chi-Square and Fisher’s Exact Chi-Square tests. In the multivariate analysis, logistic regression was used to predict the presence of chest wall deformity based on possible factors identified in previous analyses. The Hosmer-Lemeshow test was employed to assess model fit, with a *p*-value greater than 0.05 considered indicative of a good fit. Results were evaluated within a 95% confidence interval, and a *p*-value of less than 0.05 was considered statistically significant.

## Results

In our study, the mean age of the participants was 8.46 ± 5.00 years, with 76.2% being male and 23.8% female. A total of 71 patients with chest wall deformities and 80 control subjects were included. Among those with chest wall deformities, 78.9% had PE and 21.1% had PC. Additionally, 78.9% of patients with chest wall deformities were male, while 21.1% were female. The average vitamin D level among participants was 38.08 ± 16.79 ng/mL, while the mean serum Ca, P, and ALP levels were 9.82 ± 0.81 mg/dL, 5.10 ± 0.78 mg/dL, and 244.43 ± 99.80 U/L, respectively.

Among patients with chest wall deformities, 76.1% were found to have normal echocardiograms, while 15.5% had abnormal findings, and 8.4% did not undergo echocardiography. Notably, 72.7% of those with abnormal echocardiograms had PE, whereas 27.3% had PC. Furthermore, 63.6% of individuals with abnormal echocardiograms exhibited only one abnormality, while 36.4% had multiple findings. Specifically, among those with abnormal echocardiograms, 54.5% had mitral insufficiency, 45.5% had mitral valve prolapse, 27.3% had atrial septal defects, 9.1% had tricuspid insufficiency, and 9.1% had aortic insufficiency (see Table 1).

**Table 1:** Frequency distributions of various parameters

	*n*	%
**Gender**		
Male	115	76.2
Female	36	23.8
**Groups**		
Ekscavatum	55	36.4
Carinatum	16	10.6
Control	80	53.0
**Echocardiography**		
Normal	54	35.8
Abnormal	11	7.3
None	86	57.0
Echocardiography Abnormal* ( ***n*** ** = 11)**		
Mitral Regurgitation	6	54.5
Mitral Valve Prolapse	5	45.5
Atrial Septal Defect	3	27.3
Tricuspid Regurgitation	1	9.1
Aortic Regurgitation	1	9.1

Patients with chest wall deformities exhibited significantly lower levels of vitamin D (25.37 ± 11.78 ng/mL) compared to those without deformities (49.36 ± 11.76 ng/mL). Similarly, serum Ca levels were significantly lower in patients with chest wall deformities (9.36 ± 0.85 mg/dL) than in those without (10.23 ± 0.49 mg/dL). Additionally, patients with chest wall deformities had significantly lower serum P levels (4.72 ± 0.67 mg/dL) compared to those without (5.44 ± 0.71 mg/dL). While not statistically significant, patients with chest wall deformities tended to have higher serum ALP levels (257.42 ± 136.67 U/L) than those without deformities (232.90 ± 45.56 U/L). There was no statistically significant difference observed in age and gender distribution between patients with and without chest wall deformities (see Table 2).

**Table 2:** Comparison of patients with and without chest wall deformity according to various parameters

	Chest Wall Deformity(+)	Chest Wall Deformity (-)	Statistical Analysis
**Age**	8.54±5.08	8.38±4.96	t = 0.198 *p* = 0.844
**Gender**			
Male	55	60	x^2^ = 0.126 *P* = 0.723
Female	16	20	
**Vit D**	25.37±11.78	49.36±11.76	**t = -12.501** *p* < 0.001
**Ca**	9.36±0.85	10.23±0.49	**t = -7.756** *p* < 0.001
**P**	4.72±0.67	5.44±0.71	**t = -6.373** *p* < 0.001
**ALP**	257.42±136.67	232.90±45.56	t = 1.442 *p* = 0.153

*t: independent t test, * x^2^: chi-square test

Comparison between patients with PE and the control group revealed statistically significant differences in vitamin D, Ca, and P levels, with lower values observed in the PE group. However, no statistically significant difference was observed in age or gender distribution between the two groups. Similarly, no significant difference was found in ALP levels.

When comparing patients with PC to the control group, statistically significant differences were observed in vitamin D, Ca, and P levels, all of which were notably lower in the PC group. However, there was no statistically significant difference in age or gender distribution between the two groups. Additionally, no significant difference was found in ALP levels.

When comparing patients with PE to those with PC based on various parameters, including age, gender, vitamin D, Ca, and P levels, no statistically significant differences were observed.

When examining the results of logistic regression analysis for the presence of chest wall deformities, it was found that vitamin D and Ca levels exhibited a negative relationship, as did P levels. Specifically, a negative association was observed between the presence of chest wall deformities and vitamin D levels, with an estimated relative risk of 0.873. Similarly, a negative association was identified between the presence of chest wall deformities and Ca levels, with an estimated relative risk of 0.105. Additionally, a negative relationship was found between the presence of chest wall deformities and p levels, with an estimated relative risk of 0.392 (see Table 3).

**Table 3:** Logistic regression analysis results for the presence of chest wall deformity**

	RR (%95 CI) *	*p*
Age	0.930 (0.821–1.053)	0.253
Male Gender	1.001 (0.182–5.495)	0.999
Vit D	0.873 (0.827–0.921)	**< 0.001**
Ca	0.105 (0.030–0.366)	**< 0.001**
*P*	0.392 (0.157–0.980)	**0.045**
ALP	1.005 (0.996–1.014)	0.295

*RR: Estimated relative risk represented by odds ratio and 95% confidence interval

** Hosmer and Lemeshow Test was used for model fit and was found to be significant. x^2^ = 13.200 *p* = 0.105 (*p* > 0.05)

The Correct Classification Rate, a method utilized to assess model fit in the multiple logistic regression model, was determined to be 90.7%. Specifically, 91.3% of individuals without chest wall deformities were correctly classified as such, while 90.1% of those with chest wall deformities were accurately identified as having the condition.

## Discussion

While chest wall deformities occur with varying frequency in children, the male gender is consistently reported as being more frequently diagnosed in the general literature. In Turkey, multiple studies have consistently shown higher rates of PE and PC diagnoses among males [1, 11, 12]. Similarly, studies from Brazil and Iran also report a predominance of male diagnoses [13, 14]. Consistent with this literature, the present study observed that 77% of diagnosed cases were male.

The literature reveals that children with chest wall deformities can also be diagnosed with heart valve diseases through echocardiography. A retrospective review of PE surgery patients identified atrial septal defect (ASD) as the most common cardiac diagnosis, followed by ventricular septal defect (VSD) and tricuspid valve prolapse, with similar findings reported in other studies assessing postoperative evaluations [15–18]. A study involving 109 PE and PC patients reported mitral valve prolapse (MVP) in 25% of cases [19], while an ECHO study on asymptomatic PC patients identified aortic root dilatation as the most common abnormality, followed by MVP and patent foramen ovale (PFO) [20], and another study focusing on PE patients found MVP to be the most frequent diagnosis, with mitral regurgitation and tricuspid valve prolapse being less common [16]. In the present study, Mitral Regurgitation and MVP were the most common diagnoses, followed by ASD. Overall, ASD emerges as the most frequent cause requiring surgery for PE and PC, whereas mitral valve-related anomalies predominate in monitored and asymptomatic cases.

In the present study, we observed significantly lower levels of Ca, P, and vitamin D in both PE and PC cases compared to the control group. This finding is unique, as similar results have not been reported in the existing literature. Given the incomplete understanding of the etiology of these anomalies, it’s crucial to consider that aesthetic concerns might prevent children from getting adequate sunlight exposure. Parents and children should be asked about these concerns and referred to psychotherapy if needed. Evaluating the potential contribution of these factors to their development is essential. Incorporating these parameters into routine monitoring programs and providing necessary nutritional supplements, if warranted, for patients undergoing surgery or those under follow-up care, is crucial. Maintaining optimal levels of these vitamins and minerals, essential for bone development, can facilitate healthier childhood and adolescence experiences for children and significantly enhance the healing process for those undergoing surgery. Surveys questioning the behavior of avoiding sunlight should be administered to parents and patients on this issue, and behavioral change should be encouraged. It is advised to conduct prospective studies aimed at investigating the underlying causes of these deficiencies.

This study was conducted at a single center and utilized a retrospective design, which may limit the generalizability of the findings and introduce potential biases or inconsistencies in data quality. While acknowledging the limitations of conducting the study in a single center and the relatively small size of the case groups, it’s worth noting that the study’s sample size was deemed adequate when compared to existing literature on pectus cases. Unlike previous studies primarily focused on prevalence determination and operated cases, our study uniquely assessed follow-up patients and biochemical parameters, providing valuable insights into the broader spectrum of these conditions.

## Conclusion

While pectus anomalies can potentially cause cardiac or respiratory issues during childhood and adolescence, patients often prioritize aesthetic concerns. Consequently, research in this field typically focuses on surgical techniques, with study groups primarily consisting of preoperative or postoperative patients. Our study deviated from this norm by including all patients diagnosed with pectus. Our findings highlight the importance of closely monitoring calcium, phosphorus, and vitamin D levels in individuals with pectus. Ensuring that these levels remain within physiological norms can mitigate surgical complications and expedite postoperative recovery.

## Data Availability

Data will be available upon reasonable request from the corresponding author.
